# P2X7 is a cytotoxic receptor….maybe not: implications for cancer

**DOI:** 10.1007/s11302-020-09735-w

**Published:** 2020-10-04

**Authors:** Francesco Di Virgilio

**Affiliations:** Department of Medical Sciences, University of Ferrara, Ferrara, Italy

**Keywords:** Burnstock, Cancer, ATP, P2X7, Purinergic signaling, Tumor microenvironment

## Abstract

The tumor microenvironment is rich in extracellular ATP. This nucleotide affects both cancer and infiltrating immune cell responses by acting at P2 receptors, chiefly P2X7. ATP is then degraded to generate adenosine, a very powerful immunosuppressant. The purinergic hypothesis put forward by Geoff Burnstock prompted innovative investigation in this field and provided the intellectual framework to interpret a myriad of experimental findings. This is a short appraisal of how Geoff’s inspiration influenced cancer studies and my own investigation highlighting the key role of the P2X7 receptor.

## Introduction

Some years ago, at one of the Ferrara Meetings on Adenosine and Adenine Nucleotides, talking with Geoff Burnstock, I submitted to him what in my opinion were the most important features of the successful scientist. Geoff listened to me for a while, then he said: “Yes, creativity is important, scientific rigor and dedication of course, but do not forget lateral thinking.” “Lateral thinking” is an English way of speaking that is not easy to render in Italian, but that I translated for my own benefit as “always be curious and attentive of what happens outside your small courtyard.” I am sure that when he gave birth to the purinergic hypothesis Geoff would have never thought that one of the most fertile fields of application would be inflammation and cancer. Yet, the story of the P2X7 receptor (P2X7R) is here to prove that ground-breaking concepts have no boundaries; it is only up to us to grasp their revolutionary potential.

## ATP may be very dangerous on the outside of the plasma membrane

It had been shown by several investigators in the 1980s that extracellular ATP might have dire effects on cell metabolism, and even precipitate death [1–3]. Scattered reports described an intriguing, but poorly understood “permeabilizing” effect of extracellular ATP on the membrane of several cell types. In most cases, such reports were merely epiphenomenal, with the exception of the in-depth investigation carried out by Bastien Gomperts and his co-workers [1, 4], which led to postulate the existence of a specific receptor for extracellular ATP (the “ATP^4−^ receptor”) endowed with the property to trigger a reversible permeabilization of the plasma membrane, at least in rat mast cells, and possibly also in mouse fibroblasts [5]. Further work by Tom Steinberg and Sam Silverstein brought “ATP-mediated permeabilization” to the attention of the wider audience of immunologists and cell biologists [6, 7].

It was all too obvious that prolonged ATP-triggered permeabilization of the plasma membrane would eventually cause cell death, but at that time little attention was paid to this unpleasant consequence of the activation of the “ATP^4−^ receptor,” and in fact many assumed that this was a sort of an artifact with no physiological relevance. It is peculiar that, in all the papers by Bastien Gomperts, an investigator who made a fundamental contribution to the identification of receptors for extracellular ATP and the associated biological responses, no mention is ever made of the “purinergic hypothesis,” as if the famed mast cell “ATP^4−^ receptor” was something utterly unrelated to the physiological activity of extracellular ATP. On the contrary, Steinberg and Silverstein made an effort to put “ATP permeabilization” in the context of the immune response, and were momentous for the overall development of the purinergic hypothesis outside the important, but sectorial, neuropharmacological field. The studies by Silverstein and Steinberg laid the basis for bridging purinergic signaling with the most burning current issues in cell biology and immunology, i.e., cellular energy homeostasis, organelle transport, antigen presentation, and cell-mediated cytotoxicity.

## The road to discovery of the P2X7 receptor

In 1989, we made the intriguing observation that cytotoxic T lymphocytes were fully resistant to doses of extracellular ATP that were lytic to other T lymphocyte populations [8]. This observation prompted us to postulate that extracellular ATP did not exert a sort of general “unrestricted,” unselective, cytotoxic activity, but this was rather a specific effect, dependent on the expression of specific receptors or a specific metabolic state of the target cell. Reports that exposure of different cell types to variable, but usually fairly high, ATP concentrations caused efflux of intracellular ions and metabolites, and eventually killed cells, accumulated over the years, promoting the blasphemous proposal that ATP (the universal energy currency, the ubiquitous biochemical support of life) might be a cytotoxic molecule [9]. Not surprisingly, the largest part of the scientific community discarded these observations as mere laboratory artifacts with no physiological relevance whatsoever. We tried to contextualize these observations into the debated field of cell-mediated cytotoxicity, postulating that ATP might be one of the at the time elusive mediators of T cell–mediated killing [10] (see also the illuminating contemporary contribution by Michail Sitkovsky [11, 12].

In the mid-1980s, before they were cloned, P2 receptor nomenclature very much resembled what was once defined by Mike Williams a “random walk through the alphabet” (cited by Geoff Burnstock in [13]), and the ATP receptor of mast cell and lymphocytes was baptized “P2Z” [14]. Not many paid much attention to the peculiar effects of the “P2Z” receptor in inflammatory cells, except Geoff, who was intrigued by our heretical observations on the cytotoxic effects of extracellular ATP, and always encouraged our studies (see the Ciba Foundation Symposium on “P2 purinoceptors: Localization, Functions and Transduction Mechanisms” based on a proposal by Peter Illes and held in London in 1995 [15]).

A major obstacle to the acceptance of ATP-mediated plasma membrane permeabilization was its physiological meaning. Why should any cell express a “suicide receptor” activated by a supposedly ubiquitous physiological ligand? One obvious explanation put forward by many in the field was that this was very much a laboratory phenomenon, if not an artifact, with no pathophysiological significance since the extracellular ATP concentration would never reach the high concentrations needed to activate the low affinity P2Z receptor. Thus “extracellular ATP-mediated cytotoxicity” was nonsense. This disbelief was mitigated by the long-awaited molecular identification of the elusive P2Z receptor. In December 1995, I met Annmarie Surprenant at the International Workshop on “Extracellular Nucleotides: A novel and universal class of signalling molecules. From receptors to clinical functions,” organized by Georg Reiser and held in Magdeburg, Germany. After my talk, she came to me and said that she thought she “had P2Z.” A few months after, P2Z cloning was announced, and P2X7 was born [16].

## The P2X7 receptor has a major role in cancer

Although no direct in vivo measurement was still available, the belief started to build up that ATP might episodically accumulate to substantial levels in the extracellular space, especially at sites of trauma [17]. Thus, it could not be excluded that cells were exposed to high extracellular ATP concentrations under certain pathophysiological conditions. A break-through came thanks to the fundamental observations made in cancer cells by Jim Wiley. He carried out, initially in collaboration with George Dubyak, a thorough investigation of responses elicited by extracellular ATP in lymphocytes isolated from patients affected by B chronic lymphocytic leukemia, showing that these cells expressed a typical P2Z/P2X7R whose activation caused intracellular ion imbalance, redistribution of surface adhesion molecules, and lysis [18–21]. Similar results were reported about at the same time by Blanchard and co-workers in human monocytic leukemia cells [22]. Thus, many started to think that expression (or overexpression) of the P2Z receptor might be a feature of cancer cells. This belief was reinforced by a study from our laboratory showing that the P2Z/P2X7R was indeed overexpressed by B tumor lymphocytes, and that its expression correlated with severity of disease [23].

In those same years, Geoff started to pay greater attention to the possible role of the P2X7R in cancer cell physiology, and he published a study demonstrating that human melanomas express functional P2X7R which made these cells sensitive to the cytotoxic effect of the ATP analog BzATP [24]. These data reinforced Geoff’s hypothesis that the P2X7R might be a suitable target for anti-cancer therapy. Therefore, based on pioneering experiments by Rapaport [25], Geoff and his co-workers performed some of the earliest experiments aimed at testing the effects of the administration of high ATP doses to tumor-bearing mice, showing a substantial anti-tumor effect of extracellular ATP, although the receptor involved was not identified [26]. Therapeutic applications of ATP-mediated cytotoxicity in cancer cells were clearly appealing, yet we were puzzled by the paradox that cancer cells, that in principle should optimize survival strategies, overexpressed a cytotoxic, potentially deadly, receptor. Sure, it might be possible that the P2X7R in cancer cells was “uncoupled” from the intracellular death pathways (whether necrotic or apoptotic), as shown in human neuroblastoma cells [27], yet this latter mechanism, albeit possibly operative in some tumors, sounded very much like an ad hoc explanation. This reinforced the suspicion that there was more to the P2X7R than just cytotoxicity.

## The P2X7 receptor promotes growth

**Fig. 1:**
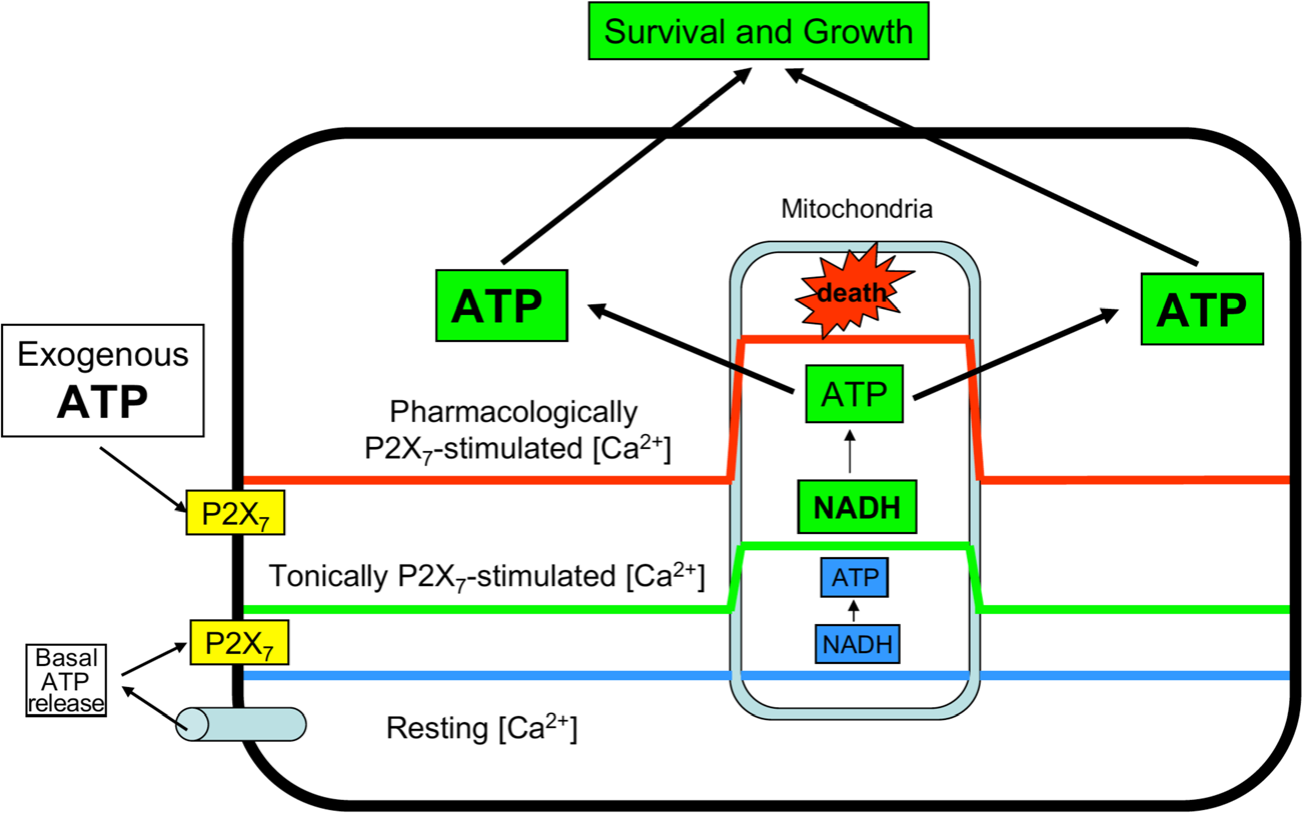
ATP is released via several plasma membrane pathways, one of which is the P2X7R itself. Thus, the P2X7R is autocrinally/paracrinally activated by ATP at different levels of stimulation: low level stimulation causes a moderate increase in the cytoplasmic Ca^2+^ concentration, which is reflected into a beneficial stimulation of mitochondrial metabolism (increased mitochondrial polarization and matrix Ca^2+^, stimulation of respiratory enzymes, enhanced ATP synthesis). On the contrary, excessive P2X7R stimulation precipitates a chain of adverse effects: mitochondrial Ca^2+^ overload, uncoupling of mitochondrial respiration, mitochondria depolarization, inhibition of ATP synthesis, and cell death. Reprinted with permission from ref. [28]

In 1996, we had reported that human T lymphocytes expressed a P2Z/P2X7-like receptor with a strong growth-promoting activity [29], and a few years later we showed that, contrary to expectations, transfection of the human P2X7R into HEK293 cells, rather than impairing survival, conferred a growth-promoting advantage [30]. We were obviously intrigued from this unexpected and counterintuitive result, and started seriously thinking that the P2X7R was not simply a cytotoxic receptor. This was not an easy hypothesis to take into consideration since we ourselves had been the most convinced advocates of the deadly “behavior” of this receptor [8, 10, 31]. I remember telling Geoff these data in several occasions and discussing with him possible interpretations, as, for example, at the Novartis Foundation Symposium “Purinergic signalling in neuron-glia interactions,” held in London in June 2005. I must say that he was not over-enthusiastic of our hypothesis: he was still convinced that ATP-dependent trophic effects were exclusively supported via P2Y receptors. However, as usual for Geoff, who never left a door closed to unorthodox results, he was not fully negative, but rather urged me to generate additional experimental data and discuss this novel evidence with him further. The turning point occurred in that same year, when we published a paper describing in detail the mechanism by which P2X7R stimulation supported mitochondrial metabolism (another heretical behavior of the P2X7R!) and promoted growth [32]. I received a phone call from Geoff congratulating for this study, at the end of which he concluded: “I have finally come round to your idea.” Thus, the concept started to consolidate that, depending on the level of activation, the P2X7R could behave alternatively as a trophic or deadly receptor (see Fig. 1).

## Preclinical studies

The demonstration that the P2X7R could promote growth provided a key to understanding the pathophysiological meaning of the P2X7R overexpression by cancer cells. In fact, tumors can exploit the P2X7R to gain advantage over healthy cells, not dissimilarly from other growth-promoting receptors. Following many in vitro demonstrations [28, 33, 34], we finally provided an in vivo proof [35]: this further convinced Geoff that we were on the right path (see [36]). It is now an established finding that several human and mouse tumors overexpress the P2X7R and that in many cases expression levels correlate with an accelerated progression [37]. To support a major role in cancer growth for the P2X7R, administration of selective blockers, synthesized by several major Pharma Industries, efficiently reduces growth and metastatization of P2X7R-expressing tumors [37–40].

Expression of the P2X7R by tumor-infiltrating immune cells adds an additional level of complication (and interest) to this picture since modulation of immune cell P2X7R contributes to the host anti-cancer response, and therefore impacts on tumor growth and metastatization [41–43]. Tumor-associated immune cells express the P2X7R to a high level and are therefore profoundly affected by extracellular ATP in the tumor microenvironment (TME). Accumulating data suggest that expression of macrophage and lymphocyte P2X7R is necessary for an effective anti-cancer immune response [41, 43], presumably because P2X7R stimulation triggers NLRP3 inflammasome activation and thus release of the potent pro-inflammatory cytokine IL-1β. This is understood to optimize tumor antigen presentation and the overall anti-tumor immune response. Accordingly, a functional P2X7R is likely needed for optimal recruitment of inflammatory cells into the tumor interstitium, as in *P2rx7*-null mice we noticed a substantial reduction of CD8^+^ T lymphocytes and an increase in Tregs [44], indicating the prevalence of an immunosuppressive microenvironment.

This is a crucial aspect of P2X7R cancer biology in view of potential therapeutic developments. It has been often objected that P2X7R targeting in cancer might be counterproductive since the anti-cancer immune response would also be impaired. However, a recent study by De Marchi et al. shows that this might not be an issue since administration of P2X7R blockers reshapes the immune infiltrate, leaving unaltered the number of CD8^+^ and Treg cells, but enhancing the number of CD4^+^ lymphocytes [44]. Interestingly, P2X7R blockade down-modulated CD39 and CD73 on CD4^+^ lymphocytes, thus alleviating immunosuppression in the TME. However, how P2X7R affects tumor-infiltrating lymphocyte (TIL) activity is not yet clear. A recent study by Grassi and co-workers shows that the P2X7R promotes TIL senescence and impairs the anti-tumor response, and accordingly lack of the P2X7R induces an enhanced anti-tumor response [45]. Selective deletion of T lymphocyte P2X7R appears to be beneficial as it reduced tumor growth and improved survival [45]. These data show that, albeit the P2X7R is an increasingly appealing target for cancer therapy, more needs to be known about the complex host-tumor interplay of which this receptor is an ineludible player. This picture is further complicated by the recent exciting finding that the P2X7R is implicated in the process of generation of CD8^+^ memory T cells [46, 47], suggesting that its blockade might hamper immunological memory.

## Purinergic signaling sheds light on the biochemistry of the tumor microenvironment

The more we know on P2X7R in cancer, the more we appreciate the importance of an in-depth knowledge of the biochemical composition of the TME. The critical point is that the P2X7R is a very low affinity, non-desensitizing, receptor [48]. This implies (a) that high concentrations of extracellular ATP are needed for its activation, and (b) that it may undergo prolonged openings in the presence of sufficient extracellular ATP concentrations. Thus, measurement of the actual ATP concentration in the TME has become critical. I remember discussing this issue with Geoff in the early days of the P2X7R; he was convinced that, although no direct measurement was as yet available, the ATP concentration in the TME was very likely high enough to drive P2X7R opening. In those days, pathways for non-lytic ATP release were still poorly characterized, but the assumption was that, at sites of inflammation (or trauma), the level of cell injury was sufficient to cause a substantial level of passive ATP leak into the interstitial milieu. Of course, the picture changed completely with the development of the pmeLUC probe that allowed a reliable semi-quantitative in vivo measurement of the extracellular ATP concentration [49–51]. Thanks to pmeLUC, we now know that ATP can accumulate in the TME to tens or even hundreds of micromoles/L, thus sufficient to drive P2X7R opening.

Other factors might also contribute to P2X7R activation in the TME. More and more data suggest that several agents may synergize with ATP at the P2X7R. Maybe, the best known is NAD^+^, another nucleotide that accumulates into the extracellular space under several pathophysiological conditions, and which, by catalyzing irreversible ADP-ribosylation of the P2X7R, lowers the Km for ATP and promotes its opening [52, 53]. However, NAD^+^ is an effective P2X7R agonist only in the mouse since human lymphocytes do not express the key ARTC2.2 enzyme necessary for ADP-ribose transfer from NAD^+^ to the P2X7R [54]. Non-nucleotide positive allosteric modulators of the P2X7R have also been identified [55–57]. Some are natural products, while others are synthesized. Some are secreted by inflammatory cells in response to bacterial infections (e.g., the cathelicidin LL-37 [58, 59]). A few drugs, e.g., polymyxin B and clemastine, were also shown to act as positive allosteric modulators of the P2X7R [60, 61]. Scattered evidence suggests that LL-37 might have a role in cancer proliferation and be a target for cancer therapy [62, 63]. Whether allosteric modulators are indeed present in the TME and modulate P2X7R activity is unknown, but this is a possibility that should be taken into consideration because on one hand it might help better understand how P2X7R function is modulated in the presence of variable level of extracellular ATP, and on the other might provide hints as how to finely tune P2X7R for therapeutic purposes.

As summarized in a recent review by Geoff Burnstock and Gill Knight, most major Pharma Industries have patented P2X7R-targeting drugs, many of which have advanced to Phase I–II clinical studies [64]. However, despite growing interest and the availability of a wealth of weapons, biologicals included, no large-scale clinical investigation on the therapeutic efficacy of P2X7R blockade in cancer has been carried out, except a small study on skin tumors [37, 65]. In this regard, the P2X7R, and in general P2 receptors, lags behind adenosine receptors which are now a major focus of attention in innovative cancer therapy [66]. Five A2A blockers are currently in clinical trials alone or in combination in cancer patients, with a good toxicity profile and encouraging preliminary results [66, 67].

## Conclusions

“Overwhelming clinical evidence supports the notion that the approach to a cancer cure must be based on targeting multiple receptors and pathways, and that the best results are obtained when physiological mechanisms for cancer cell elimination are seconded (as in the case of immunochemotherapy) rather than being ignored.” This a quote from a review on purinergic signaling and cancer co-authored with Geoff some time ago [36]. It is impressive how clearly some of the most important principles of modern cancer therapy were stated: (a) combination therapy, (b) exploitation of the host own resources for cancer cell elimination. These are the basis for the current use of immune check-point blockers and immunogenic cell death inducers [68]. The discovery that extracellular ATP is abundant in the TME and that tumor and infiltrating inflammatory cells express a variety of receptors for ATP and its metabolites (chiefly adenosine) has opened unanticipated perspectives of intervention in cancer therapy. In principle, it will be possible to prevent ATP release into the TME, thus reducing P2 receptor stimulation and adenosine generation, or target selected receptors (i.e., P2X7R or A2A) on the host or on the tumor cells. This endows purinergic signaling in cancer with an unsurpassed plasticity compared with other signal transduction systems and fuels hopes for development of novel and more effective cancer therapies for the benefit of mankind.
